# Development and evaluation of two web-based interventions for the promotion of physical activity in older adults: study protocol for a community-based controlled intervention trial

**DOI:** 10.1186/s12889-017-4446-x

**Published:** 2017-05-25

**Authors:** Saskia Muellmann, Inna Bragina, Claudia Voelcker-Rehage, Eric Rost, Sonia Lippke, Jochen Meyer, Jochen Schnauber, Merlin Wasmann, Merle Toborg, Frauke Koppelin, Tilman Brand, Hajo Zeeb, Claudia R. Pischke

**Affiliations:** 1 Leibniz Institute for Prevention Research and Epidemiology – BIPS, Achterstrasse 30, 28359 Bremen, Germany; 2 Institute of Human Movement Science and Health, Technical University Chemnitz, Chemnitz, Germany; 3 Jacobs University Bremen, Bremen, Germany; 4 OFFIS – Institute for Information Technology, Oldenburg, Germany; 5 Jade Hochschule, Oldenburg, Germany; 6 Health Sciences Bremen, University of Bremen, Bremen, Germany

**Keywords:** Physical activity, Older adults, eHealth, Intervention, Physical activity promotion, Primary prevention, Healthy ageing

## Abstract

**Background:**

Regular physical activity (PA) is a key contributor to healthy ageing. However, despite known health benefits, only one third of older adults in Germany reach the PA levels recommended for persons aged 65 years and above by the World Health Organization. The aim of the current study is to evaluate the effectiveness of two web-based interventions for the initiation and maintenance of regular PA (i.e., intervention groups 1 and 2) compared to a delayed intervention control group of older adults aged 65 to 75 years.

**Methods/Design:**

Study participants will be randomly assigned to one of three study arms in five communities in the Bremen-Oldenburg metropolitan region: a) Participants in the first arm will receive access to a web-based intervention for 10 weeks allowing them to track their weekly PA (subjective self-monitoring, intervention group 1); b) participants in the second arm will receive access to the web-based intervention for 10 weeks and, in addition, track PA using Fitbit Zips (objective self-monitoring, intervention group 2); c) participants in the delayed intervention control group will receive access to the intervention implemented in the first study arm after completion of the 12-week follow-up in the other two groups within each community. In addition, weekly group meetings in the communities will be offered to study participants in the intervention groups providing the opportunity to address questions related to the use of the website and to practice PA in groups (e.g., neighborhood walks, strength and balance exercises). To evaluate short-term effects of the intervention on physical and psychological health, PA, physical fitness, and cognitive and psychological variables will be assessed at baseline and 12-week follow-up.

**Discussion:**

This study will provide answers regarding acceptance and effectiveness of web-based interventions promoting uptake and maintenance of regular PA in persons aged 65–75 years. Study findings will contribute to a growing body of evidence in Germany concerning the role of community-based interventions for the promotion of PA and healthy ageing in older adults.

**Trial registration:**

German Clinical Trials Register DRKS00010052 (Date of registration 07–11-2016).

## Background

Regular physical activity (PA) is associated with improvements in physical, psychological, cognitive, and functional health [1–4]. The World Health Organization (WHO) and the American College of Sports Medicine (ACSM) recommend a weekly moderate exercise time of 150 min for adults aged 60 years and above. Moreover, it is recommended that older adults engage in flexibility and strength training at least two times per week [5, 6]. The percentages of older adults (aged 60 years and above) meeting the recommendation for moderate exercise time range from 2% to 83%, depending on the study [7]. In Germany, only 18% of adults between the ages of 60 and 69 years and 14% of adults between the ages of 70 and 79 years meet the current recommendations for PA [8].

Physical limitations due to health conditions or age-related restrictions, as well as a lack of age-appropriate PA programs and information regarding access to such programs, are barriers to program participation and to reaching PA recommendations in this population [9]. In contrast, high levels of personal motivation to stay physically and mentally active, as well as having access to affordable and appropriate exercise options, are perceived as facilitators for reaching and maintaining PA recommendations by older adults [9].

Interventions providing information on PA as print versions [10, 11] or face-to-face [12] have a long tradition and previous studies suggests that these interventions are effective in promoting PA in older adults. The increased use of the internet and mobile technologies in recent years may open up new opportunities for promoting PA in this population [13, 14]. In Germany, 50% of adults aged 60 years and above already use the internet regularly [15], 17% of adults aged 65 years and above use smartphones [16], and this trend is increasing. Hence, eHealth interventions (i.e., measures to promote health using information and communication technologies [17]), appear promising for reaching this population and for providing individualized PA programs. To date, a variety of studies (predominantly conducted outside of Germany) investigated the role of eHealth interventions to promote PA in older adults suggesting that participation in eHealth interventions leads to increased levels of PA [18–21].

Because a large body of evidence indicates that tailoring of intervention materials and messages to participants’ characteristics, such as gender, PA level at study entry, and readiness to engage in PA, is associated with greater success in achieving PA goals in the long-term [20, 22–26], intervention materials employed in the majority of the interventions in the above cited studies used tailoring. However, although a wide range of PA interventions is available in Germany [27], only few are tailored to characteristics and needs of older adults or readiness to engage in PA and the majority of studies investigating effects of PA interventions in this population demonstrated rather small behavioral changes [25]. Furthermore, the effects of self-monitoring of PA behavior via web-based diaries, logs or tracking devices have, thus far, not been systematically investigated in older populations in Germany.

Therefore, the main aim of the current study is to compare the effectiveness of two different web-based interventions (encouraging subjective vs. subjective and objective PA self-monitoring) among older adults living in five communities in the Bremen-Oldenburg metropolitan region to a delayed intervention control group. Recommendations regarding PA and intervention materials provided to participants of both web-based interventions will be tailored to gender, PA-level, and stage of readiness to engage in the interventions assessed at baseline. The study is embedded in the larger Physical Activity and Health Equity: Primary Prevention for Healthy Ageing (AEQUIPA) research network which is funded by the Federal Ministry of Education and Research (BMBF).

## Methods

### Aims of the overall AEQUIPA project and network

The network conducts theory-based and participatory empirical research in the Northwestern part of Germany (http://www.aequipa.de/en/home.html, [28]). It aims to develop, implement, and evaluate PA interventions for the primary prevention of chronic diseases in persons aged 65 years and above. With five subprojects, AEQUIPA’s goal is to strengthen the evidence base for preventive PA in the context of healthy ageing and to gain new insights into environmental, social-contextual, and individual factors influencing PA in persons aged 65 years and above. This study is one of five subprojects of the entire network.

### Study aims

The following main research question will be examined in this study:

- Is a web-based intervention with subjective and objective PA monitoring more effective for the promotion of PA among older adults than a web-based intervention with subjective PA monitoring only compared to a delayed intervention control group?

Secondary research questions of the study are the following:

- Do participants’ characteristics (e.g., age, gender, motivational stage) influence intervention attendance and PA behavior?
- Is participation in the web-based interventions associated with improvements in secondary outcomes, such as well-being, quality of life, fear of falling, physical and cognitive functioning?

An additional objective of the study is to improve our understanding of how web-based interventions and technologies for PA tracking ought to be designed to be usable for the target group and which features ought to be included from the user’s point of view.

### Selection of communities for the study

Preceding recruitment for this study and as part of another subproject of the AEQUIPA network (RTC project, see [29]), a cross-sectional community readiness assessment (CRA) regarding the uptake and/or implementation of PA interventions in older adults (65–75 years) was conducted in a sample of municipalities within the Bremen-Oldenburg metropolitan region. The CRA was based on a structured interview administered to key informants, such as representatives from local public authorities, senior citizen organizations or sports clubs in each of the selected communities [29]. Municipalities were selected for inclusion if they already had a comparably high proportion of older adults or if they expected a high increase in the proportion of older adults living in the region over the next decade. Overall, 23 municipalities (12 rural, 11 urban) were included in the assessment. The five communities with the lowest levels of community readiness were selected for the implementation of PA interventions (three urban communities: Burglesum, Bremen; Vahr, Bremen; Obervieland, Bremen; two rural communities: Osterholz-Scharmbeck, Lower Saxony; Achim, Lower Saxony).

### Participants and procedures

Names and addresses of men and women between the ages of 65 and 75 years residing in these five communities of the metropolitan region of Bremen-Oldenburg will be drawn from the records of the residents’ registration office. Subsequently, persons will be invited to participate in the study via mail. The study will also be publicized in local newspaper articles, in senior organizations, and as part of capacity building activities of the RTC project. Eligibility for study participation will be determined in telephone interviews with trained study nurses following the inclusion and exclusion criteria outlined below.

#### Inclusion and exclusion criteria

Residents of the five communities will be eligible for study participation if they are between the ages of 65 and 75 years, if they are able to live independently (i.e., in own apartment or room without assisted living, no regular home nursing), and if they provide an informed consent to participate in the study. Further criteria for inclusion in the study are basic knowledge of German, the ability to walk without a walking aid, and to participate in study assessments and weekly group meetings without external support. Also, internet access at home or at family members’ or friends’ houses is a precondition for participation. Participants will be excluded from the study if they have planned a vacation for more than one month during the intervention period, display cognitive impairment (Mini-Mental-Score ≤ 27) or other permanent impairments (e.g., stroke, neurological diseases, such as Parkinson’s) or if there are any medical contraindications regarding program participation.

**Fig. 1:**
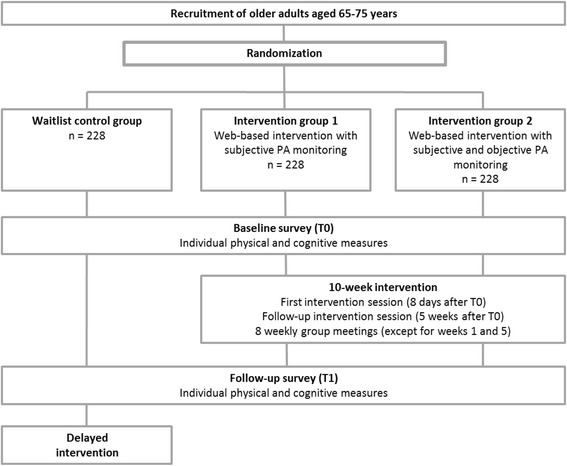
Study Design

After successful screening for study eligibility, study participants will be assigned to one of three study arms by the study nurses: a) a web-based intervention with subjective PA self-monitoring (intervention group 1), b) a web-based intervention with subjective and objective PA self-monitoring (intervention group 2) or c) a delayed intervention control group (waitlisted control group) receiving the intervention of intervention group 1 after completion of the 12-week follow-up (see Fig. 1 for the study design). Each intervention condition will be randomly assigned to certain weeks of baseline assessment. Participants will be free to choose from available time slots during a phone call with a study nurse, but only after their decision will they be informed about which intervention condition was assigned to this particular week.

### Measures

Participants will be invited to the study center to complete baseline (T0) and follow-up assessments (T1). At the study center, participants will undergo different anthropometric, physical, motor and cognitive tests, as well as an assessment of motivational stages to engage in PA. Cardiovascular fitness will be assessed using the 2-min step test [30]. Functional status and physical performance will be measured employing the Short Physical Performance Battery (SPPB) which includes balance, gait speed, and chair raising tests [31]. Moreover, postural control under dynamic conditions [32], strength of the upper extremities [33], hand grip strength [34], height, weight, and body fat will be assessed using stadiometers and bioimpedance scales. Cognitive dysfunction [35], memory (Verbal Learning and Memory Test (VLMT), [36]), and attention and inhibition (Simon Task, [37]) will be assessed in cognitive tests. In addition, working memory updating and inhibition (Random Number Generation Test (RNGT), [38]), and postural control under static conditions (measure of tandem stand by use of iPad, software: Sensor Data, Wavefront Labs, [39]) will be assessed under single- and dual-task conditions. Motivational stages regarding PA will be measured via a validated algorithm [40, 41].

After both assessments, all participants will receive an accelerometer (ActiGraph GT3x+) to objectively measure PA and a self-administered questionnaire. Participants will be instructed to wear the accelerometer on the right hip and during the day for seven days following baseline and follow-up assessments. The self-administered questionnaire will contain validated instruments and self-generated items assessing levels of PA, perceived physical environment, social support, health behavior, quality of life, and previous experience with using technology (for further detail on the instruments included in the questionnaire, see Table 1). Sociodemographic information will include age, gender, migration background, education, employment, and household income. In addition, participants of intervention groups 1 and 2 will fill out a self-administered questionnaire at the follow-up comprised of self-generated items regarding use and acceptance of the website, attendance of the offered group sessions, and overall satisfaction with the interventions.

**Table 1:** Measures in the self-administered study questionnaire

Outcome measure	Instrument/scale
Physical activity	
Physical activity	International Physical Activity Questionnaire (IPAQ) [49]
Intention to engage in physical activity	Health Action Process Approach (HAPA), intention [40, 41]
Self-efficacy regarding physical activity	HAPA, self-efficacy [40, 41]
Planning for physical activity	HAPA, planning [40, 41]
Physical self description	Physical self-description (PSDQ) [50]
Physical environment	
Physical activity and neighborhood environment	International Physical Activity Questionnaire Environmental module (IPAQ-E) [51]
Social support, social activities	
Social support for engaging in physical activity	Social support and exercise survey (modified, [40, 41])
Social networks	Self-generated items
Social activities	Florida Cognitive Activities Scale (modified, [52, 53])
Health behavior	
Subjective age	Self-generated item
Health-related quality of life	Short-Form (SF)-12, only 1 item [54]
Objective health	Diseases and medication use (modified, [55])
Risk perception	Berlin Risk Appraisal and Health Motivation Study (BRAHMS) [56]
Falls	Elderly Fall Screening Test (EFST) (modified, [57])
Fear of falling	Geriatric Fear of Falling Measurement (GFFM) [58]
Diet	Food Frequency Questionnaire [59]
Alcohol consumption	Alcohol Use Disorders Identification Test Short Version (AUDIT-C) [60]
Smoking behavior	Smoking Behavior Questionnaire [61]
Stage assessment of smoking behavior, alcohol consumption, fruits and vegetable consumption	Stage assessment [40, 41]
Quality of life and well-being	
Quality of life	Satisfaction with Life Scale (SWLS) [62]
Emotional well-being	Self-generated items
Depression	Centers for Epidemiologic Studies Depression Scale (CES-D) [63]
Personality	NEO Five Factor Inventory (NEO-FFI) [64]
Previous experiences with technology	
Use of computers/smartphones/applications	Self-generated items
Technology commitment	Technology Commitment Scale [65]
Use, acceptance, and satisfaction with interventions	
Use and acceptance of various components of the website, attendance of the offered group sessions, and overall satisfaction with the interventions	Self-generated items

### Interventions

Two web-based interventions promoting self-monitoring of PA will be developed. Both will be based on self-regulation theory [42, 43] and on principles of behavior change (e.g., shaping knowledge, feedback and monitoring, goals and planning, social support, comparison of behavior, rewards, [44]). Participants in the intervention groups will receive brochures with PA recommendations including exercises to improve balance (two times per week), strength (on two or more nonconsecutive days per week involving major muscle groups), and endurance (for at least 150 min with moderate intensity or at least 75 min with vigorous intensity each week in bouts of 10 min, or an appropriate combination of both types of activities), according to the recommendations of the WHO and the ACSM. Depending on PA-level assessed at baseline and gender, participants will be provided with different brochures outlining exercises for different levels of difficulty and displaying pictures of male vs. female older adults modeling the exercises.

In the first intervention arm (intervention group 1), participants will receive access to a web-based PA diary and will be encouraged to track their behavior over a 10-week period. Participants in the second intervention arm (intervention group 2) will additionally receive Fitbit Zips (Fitbit, San Francisco, USA) to objectively track PA; data of the Fitbit Zips will be synchronized with the website following regular time intervals. The website will provide weekly feedback on whether PA goals (WHO recommendations for moderate exercise time, flexibility and strength training) are reached (and goal-specific rewards), and will provide opportunities to network with other intervention participants via an invite friends function and a forum. In addition to the web-based interventions, participants will be offered weekly group meetings in their communities led by trained research assistants. During these 90-min meetings, participants can resolve technical problems with the website, receive health education regarding healthy ageing, and practice PA in groups. All intervention materials (e.g., PA recommendations and instructions) will be tailored to participants’ age, gender, motivation to engage in PA, and PA-level assessed at baseline.

One week after the baseline assessment, the intervention will be introduced to participants of intervention groups 1 and 2 separately in group sessions with a maximum of 24 participants. Five weeks after the start of the intervention, a second group meeting will be held to ensure the proper use of the website and the Fitbit Zips in the two intervention groups. After completion of the follow-up assessment, persons in the delayed intervention control group will receive access to the web-based intervention of intervention group 1. However, no weekly group meetings will be offered to participants in this study arm.

### Qualitative research informing the design of the interventions

All assessment and intervention materials will be pilot tested with seniors in a different region of Germany than the intervention and assessment sites to prevent spill-over effects. A four-week pilot intervention and a focus group interview will be conducted. During this pilot, participants will be asked whether they are satisfied with the support received during the intervention period and with the comprehensibility and difficulty of the exercise brochures. Focus groups with participants who receive Fitbit Zips to objectively track PA in the second intervention arm will be conducted to assess the usability of the Fitbit Zips. Adaptations to all intervention materials and the website will be made based on the results of these pilot tests.

### Analytic strategy

#### Quantitative analysis, sample size

Hierarchical linear regression models will be used to analyze the intervention effects. Due to the week-wise randomization scheme, recruitment week will be included as a clustering variable. Change over time in objectively measured PA will serve as the primary outcome variable. Change over time will be calculated by subtracting the baseline value from the follow-up value (PA_T1_-PA_T0_). To assess the intervention effects, two dummy variables for intervention groups 1 and 2 will be added contrasting the change over time in PA in each intervention arm with the changes in the delayed intervention control group. Expecting small to moderate intervention effects [45], we calculated the sample size assuming a standardized mean difference of 0.33 in change over time between the intervention groups and the delayed intervention control group. Further, assuming ten clusters (recruitment weeks) per study arm and an intraclass correlation of 0.01, 190 participants per study arm will be necessary for the analysis with α = 0.05 (two-sided test) and β = 0.20. Expecting a loss to follow-up of 20%, we aim for a sample size of *n* = 684 at baseline.

Changes in cardiovascular fitness, physical performance, body fat, cognitive dysfunction, fear of falling, and quality of life will be analyzed as secondary outcomes. Several variables will be included as potential confounders, such as baseline PA-level, age, gender, education, motivational stage, and other health behaviors. In additional analyses, the moderating influence of gender, socioeconomic strata, and motivational stage on intervention effects will be explored.

#### Qualitative analysis

Qualitative focus group discussions (eight groups) will be protocolled by two researchers and audio-recorded. The aim of these focus groups discussions will be to assess the usability of the web-based intervention with subjective and objective PA monitoring (intervention group 2). Results of these focus groups will form the basis for improving interventions in the future, particularly for different user groups (e.g., females). Audio-records will be transcribed and records will be compared and complemented by analyzing the protocols. Both, deductive and inductive methods will be used for analyzing the data based on Mayring [46].

### Ethics statement and consent

This study was approved by the Ethics Committee of the Technical University of Chemnitz (TU Chemnitz), Faculty of Behavioural and Social Sciences, on July 14, 2015 – number V-099-17-HS-CVR-PROMOTE-03072015. The study was registered at the German Clinical Trials Register on July 11, 2016 – number DRKS00010052. All study participants will be fully informed about the study and will be requested to give informed consent.

### Expected results

We expect to find more pronounced increases in PA and the secondary outcomes in the two intervention arms (intervention groups 1 and 2) compared to the delayed intervention control group, as well as more pronounced intervention effects in persons in intervention group 2 compared to intervention group 1. We base this assumption on previous trials conducted in the United States and other parts of Europe demonstrating significant increases in PA in persons participating in web-based interventions for PA promotion compared to control groups (e.g., [18, 21, 26, 47, 48]).

## Discussion

This study will provide answers regarding acceptance and effectiveness of web-based interventions for the promotion of PA in persons aged 65–75 years living in Germany. Study findings will be interpreted alongside the results obtained in the other subprojects of the AEQUIPA project and network, hence contributing to the multi-disciplinary evidence regarding the relationship of PA and health and well-being among seniors.

## Abbreviations

ACSM: American College of Sports Medicine
AEQUIPA: Physical Activity and Health Equity: Primary Prevention for Healthy Ageing
AUDIT-C: Alcohol Use Disorders Identification Test Short Version
BMBF: German Federal Ministry of Education and Research
BRAHMS: Berlin Risk Appraisal and Health Motivation Study
CES-D: Centers for Epidemiologic Studies Depression Scale
CRA: Community Readiness Assessment
EFST: Elderly Fall Screening Test
GFFM: Geriatric Fear of Falling Measurement
HAPA: Health Action Process Approach
IPAQ: International Physical Activity Questionnaire
IPAQ-E: International Physical Activity Questionnaire Environmental module
NEO-FFI: NEO Five Factor Inventory
PA: Physical activity
PSDQ: Physical self-description
RNGT: Random Number Generation Test
SF: Short-Form
SPPB: Short Physical Performance Battery
SWLS: Satisfaction with Life Scale
VLMT: Verbal Learning and Memory Test
WHO: World Health Organization
